# Skraban-Deardorff intellectual disability syndrome-associated mutations in WDR26 impair CTLH E3 complex assembly

**DOI:** 10.1002/1873-3468.14866

**Published:** 2024-04-04

**Authors:** Annette Gross, Judith Müller, Jakub Chrustowicz, Alexander Strasser, Karthik V. Gottemukkala, Dawafuti Sherpa, Brenda A. Schulman, Peter J. Murray, Arno F. Alpi

**Affiliations:** 1 Immunoregulation Research Group, Max Planck Institute of Biochemistry, Martinsried, Germany; 2 Department of Molecular Machines and Signaling, Max Planck Institute of Biochemistry, Martinsried, Germany

**Keywords:** Skraban-Deardorff syndrome, E3 ubiquitin ligase, intellectual disability syndrome, SKDEAS, GID E3 complex, CTLH E3 complex, WDR26, YPEL5, HBP1

## Abstract

Patients with Skraban-Deardorff syndrome (SKDEAS), a neurodevelopmental syndrome associated with a spectrum of developmental and intellectual delays and disabilities, harbor diverse mutations in *WDR26*, encoding a subunit of the multiprotein CTLH E3 ubiquitin ligase complex. Structural studies revealed that homodimers of WDR26 bridge two core-CTLH E3 complexes to generate giant, hollow oval-shaped supramolecular CTLH E3 assemblies. Additionally, WDR26 mediates CTLH E3 complex binding to subunit YPEL5 and functions as substrate receptor for the transcriptional repressor HBP1. Here, we mapped SKDEAS-associated mutations on WDR26 structural model and tested their functionality in complementation studies using genetically engineered human cells lacking CTLH E3 supramolecular assemblies. Despite the diversity of mutations, fifteen of sixteen tested mutants impaired at least one CTLH E3 complex function contributing to complex assembly and interactions, thus providing first mechanistic insight into SKDEAS pathology.

## Abbreviations

ARID1B: AT-Rich Interaction Domain 1B
ARMC8: Armadillo Repeat Containing 8
CRA: CT11-RanBPM
CTLH: C-terminal to LisH
CUL3-RBX1: Cullin 3 Ring-Box 1
FADD: Fas-associated Death Domain
GID: Glucose-Induced Degradation Deficient
GPCR: G-protein Coupled Receptor
HBP1: HMG-Box Transcription Factor 1
Ind: Individual
LisH: Lissencephaly type-1 Like Homology Motif
MAEA: Macrophage Erythroblast Attacher
MKLN1: Muskelin
PD-L1: Programmed Death-Ligand 1
RANBP9: RAN Binding Protein 9
RMND5A: Required For Meiotic Nuclear Division 5 Homolog A
SKDEAS: Skraban-Deardorff Syndrome
SPOP: Speckle Type BTB/POZ Protein
STR: Short Tandem Repeat
TET2: Ten-Eleven Translocation-2
TWA1: Two Hybrid Associated Protein 1 With RanBPM
WD40: WD40 Repeat Domain
WDR26: WD Repeat Containing Protein 26
YPEL5: Yippee Like 5
ZMYND11: Zinc Finger MYND-type Containing 11

## Introduction

Skraban-Deardorff syndrome (SKDEAS, OMIM: #617616) is a recently discovered neurodevelopmental syndrome associated with a spectrum of facial dysmorphias, developmental and intellectual delays and disabilities, gum diseases and other oral-cranial manifestations [1]. The genetics of SKDEAS center on mutations in *WDR26*. In most SKDEAS patients described so far, the *WDR26* region (1q41-q42) contains additional microdeletions that may affect other genes in the region [1–8]. Nevertheless, *WDR26* mutations are common to all SKDEAS patients, and thus a reasonable hypothesis is that mutation of *WDR26* is causative for the pathophysiological manifestations of the syndrome. Notably, SKDEAS *WDR26* mutations encompass a variety of deletions, frame shifts, and single missense mutations. A key question therefore is, how each *WDR26* mutation contributes to SKDEAS.

*WDR26* encodes a subunit of the multiprotein C-terminal to LisH (CTLH) E3 ubiquitin ligase complex. The CTLH E3 is the mammalian orthologue of the *S. cerevisiae* glucose-induced degradation deficient (GID) complex [9]. Both the CTLH and GID E3 complexes refer to a collection of various large multiprotein assemblies that target specific proteins for degradation [10–13]. The best understood members of the GID/CTLH E3 family are from *S. cerevisiae*. Yeast GID E3 recruits metabolic enzyme substrates to interchangeable “Gid4-like” family substrate receptors (Gid4, Gid10 and Gid11) for their degradation in response to metabolic stress cues [10, 11, 14–19]. In contrast, humans encode only one Gid4-like substrate receptors, GID4, that has been implicated in binding a plethora of cellular proteins, at least some of which are presumably CTLH E3 ligase substrates [11, 20–28]. Alternative to GID4, WDR26 was proposed to also serve as a substrate receptor [21]. WDR26 mediates association with the transcriptional repressor HBP1 and was shown to effect cellular HBP1 protein turnover [21, 29]. Besides, structural studies suggest direct binding of WDR26 dimers with the CTLH E3 complex subunit YPEL5, however, the functional role of this interaction remains to be uncovered. Although yeast harbors an ortholog of human YPEL5 [30], at this point it is unknown whether it binds the yeast orthologue of WDR26, Gid7, or if Gid7 could mediate substrate-targeting in some GID E3 assemblies.

**Fig. 1:**
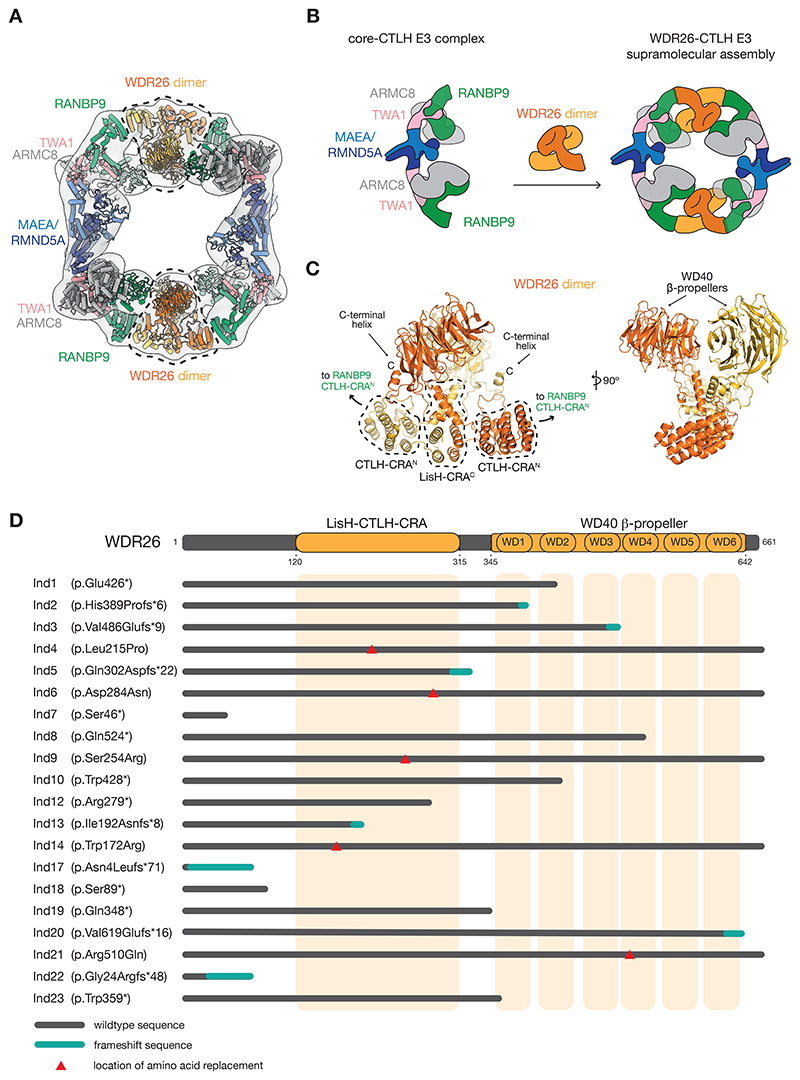
Supramolecular assembly and domain architecture of WDR26 mutants. A) 19.2-Å-resolution cryo-EM map of the WDR26-CTLH complex (EMD-12542) composed of WDR26, RANBP9, TWA1, ARMC8, MAEA and RMND5A at a 2:2:2:2:1:1 ratio. The map was fitted with AF models of WDR26 and the catalytic module as well as the prior cryo-EM structure of the scaffolding module (PDB: 7NSC). CTLH subunits are color-coded as follows: MAEA, blue; RMND5A, dark blue; TWA1, pink; RANBP9, green; ARMC8, grey; WDR26, orange and yellow. B) Cartoon illustrating how WDR26 dimers connect two core CTLH E3 complexes into a supramolecular WDR26-CTLH E3 assembly. The CTLH subunits are color-coded as in A). C) AF model of a dimer of WDR26 isoform 1 (UniProt ID: Q9H7D7-1) highlighting its functionally important parts. The LisH-CRA^C^ domains, edges of the WD40 β-propeller, and the C-terminal helices contribute to WDR26 dimerization, whereas the CTLH-CRA^N^ domains interact with the corresponding CTLH-CRA^N^ domains of RANBP9 within the neighboring scaffolding modules. D) List of pathogenic WDR26 variants indicating their positions in the WDR26 domain schematic highlighting the LisH-CTLH-CRA unit and WD40 β-propeller (comprising six WD40 motifs WD1-6). Locations of single amino acid substitutions (red triangle), wild type sequence (dark gray) and frameshift sequences (turquoise) are indicated.

Nonetheless, human WDR26 and yeast Gid7 have a common function: mediating formation of higher-order GID/CTLH E3 assemblies. WDR26 homodimers bridge two core-CTLH E3 complexes to generate a giant, hollow oval-shaped supramolecular WDR26-CTLH E3 complex (Fig. 1A and 1B) [11]. WDR26 self-assembly can be modulated by binding RANBP9, which is a subunit of the scaffolding module serving as a bridge between WDR26 and other CTLH E3 ligase subunits (Fig. 1B) [11, 21, 31]. Higher eukaryotes have another Gid7 ortholog called Muskelin (encoded by *MKLN1*), which itself can form oligomers and also mediates supramolecular assembly of distinct CTLH E3 complexes [11, 31–33]. As such, higher eukaryotes express multiple CTLH E3 assemblies [11, 31, 33, 34]. Indeed, several studies indicate that various CTLH E3 complexes have a range of functions [12, 13, 35], including cell proliferation, differentiation and neuronal development [1, 29, 36–41]. In particular, WDR26 has been associated with a variety of biological processes including G protein-coupled receptor (GPCR) signaling [35, 42–47], Wnt signaling [48, 49], nuclear condensation, and erythropoiesis [50, 51]. However, it is currently unclear whether supramolecular WDR26-CTLH E3 assemblies are involved in these processes.

Despite the recent progress in understanding the structural assembly of the WDR26-CTLH E3 complexes, how these are affected by SKDEAS WDR26 mutations remains unknown. Here, we assayed properties of diverse WDR26 mutations on forming cellular CTLH E3 assemblies, and binding of the WDR26-associated proteins YPEL5 and HBP1. Our findings reveal all tested mutations, except one, cause defects in WDR26-CTLH E3 higher-order assembly, formation of a complex with CTLH E3 scaffolding and catalytic subunits, and/or binding to interacting proteins, thus implicating impaired CTLH E3 activities in SKDEAS patients.

## Material and methods

### Maintenance and Generation of Cell Lines

K562 erythroleukemia cell line was originally purchased from ATCC (CCL-243™). K562 cells were cultured in IMDM (Gibco), supplemented with 10% fetal bovine serum (FBS, Gibco), 1x Penicillin& Streptomycin (VWR) and 1x GlutaMax (Sigma) at 37°C and 5% CO_2_. To generate stable cell lines, cells were transfected with Lipofectamine 3000 (Sigma) using 5 µg of plasmid DNA, according to the manufacturer’s protocol. Cells were then continuously cultured in the presence of 200 µg/mL Hygromycin B (Thermo Fisher Scientific). *WDR26*^-/-^; *MKLN1*^-/-^ double knockout K562 cells were already described previously [11]. Flp-In T-Rex-HEK293 cells were obtained from Thermo Fisher Scientific and cultured in DMEM (Gibco) supplemented with 10% FBS (Gibco), 1x Penicillin& Streptomycin (VWR), 15 µg/mL Blasdicidin S HCl (Gibco), 100 µg/mL Zeocin (Gibco) and 1x GlutaMax (Sigma) at 37°C and 5% CO_2_. All cell lines are authenticated by STR and were constitutively tested negative for mycoplasma infection by PCR.

*WDR26*-deficient HEK293 cells were generated using CRISPR-Cas9 (D10A) nickase genome editing strategy. ATUM gRNA design tool was utilized to identify paired sense and antisense guide RNAs (gRNA) targeting exon 1 of *WDR26* as described recently [11]. Sense and antisense gRNA were cloned into pBABED-U6-Puromycin plasmid (gift from Thomas Macartney, University of Dundee, UK) and pX335-Cas9 (D10A) (Addgene), respectively. Flp-In T-Rex-HEK293 cells were co-transfected with sense/antisense gRNA plasmids using Lipofectamine 3000 transfection reagent (Thermo, Invitrogen). 24 hr after transfection, cells were selected in puromycin (2 µg/ml) for two days, followed by expansion, and single-cell dilution to obtain cell clones. Successful knockout clones were verified by immunoblotting and genomic sequencing of targeted loci.

### Generation of HA-WDR26 and mutated versions

Human *WDR26* isoform 1 was used as reference template and was synthesized with an N-terminal HA-tag and the cDNA regions 18-117 and 156-366 were codon-optimized due to a very repetitive GC-rich sequence (Twist Bioscience). Human *WDR26* isoform 2 was synthesized with an N-terminal HA-tag and codon-optimized (Twist Bioscience). The cDNA was cloned into a pcDNA3.1 backbone encoding hygromycin resistance. Primers for site directed mutagenesis were designed with the QuickChangeII site-directed mutagenesis tool (Agilent) and introduced using Turbo-Pfu polymerase according to the manufacturer’s protocol (Agilent). PCR products were then amplified using XL1 blue competent *E. coli* and confirmed by sequencing (Eurofins Genomics). Mutagenesis primers are the following:

Ind1 (Fwd: caaagccaaagctaagagcagtcatctgggcc, Rev: ggcccagatgactgctcttagctttggctttg),

Ind2 (Fwd: cagttttagcaggtgtgtccggatcaacttgcca, Rev: tggcaagttgatccggacacacctgctaaaactg),

Ind3 (Fwd: ggcattgcactcttccccttcccaggag, Rev: ctcctgggaaggggaagagtgcaatgcc),

Ind4 (Fwd: ggtacttctgctgcagcggcaaaaacttcatcctc, Rev: gaggatgaagtttttgccgctgcagcagaagtacc),

Ind5 (Fwd: cgcaggagagtctaaacgccgtggggg, Rev: cccccacggcgtttagactctcctgcg),

Ind6 (Fwd: ggtaaataggtctgaagtttattcaatagtttagatcgggaagct, Rev: agcttcccgatctaaactattgaataaacttcagacctatttacc),

Ind7 (Fwd: gcggacgacgactacgagggggacg, Rev: cgtccccctcgtagtcgtcgtccgc),

Ind8 (Fwd: taggatgatcttcttatactatgttcctatctgtaaggtcctcg, Rev: cgaggaccttacagataggaacatagtataagaagatcatccta),

Ind9 (Fwd: ctacacatcagataccccctaagaacatgaatgcgc, Rev: gcgcattcatgttcttagggggtatctgatgtgtag),

Ind10 (Fwd: ctgtttgtacattccaaagtcaaagctcagagcagtcat, Rev: atgactgctctgagctttgactttggaatgtacaaacag),

Ind12 (Fwd: ttatccaatagtttagatcaggaagctgtccctttgcc, Rev: ggcaaagggacagcttcctgatctaaactattggataa),

Ind13 (Fwd: agtgcattctcctcatgctaattgtggtaagaggcg, Rev: cgcctcttaccacaattagcatgaggagaatgcact),

Ind14 (Fwd: cattttctgccttatccctgtctccttccatgacatg, Rev: catgtcatggaaggagacagggataaggcagaaaatg),

Ind18 (Fwd: cgccacagtggcttaggacgcggctgctg, Rev: cagcagccgcgtcctaagccactgtggcg),

Ind19 (Fwd: gctccgtaagtatctgctacgtataacatgggaactg, Rev: cagttcccatgttatacgtagcagatacttacggagc),

Ind20 (Fwd: atctgtgggttccagctcacagtttactgtacgt, Rev: acgtacagtaaactgtgagctggaacccacagat),

Ind21 (Fwd: ctcgaagttatagccctgaattcgctggtgtgtatc, Rev: gatacacaccagcgaattcagggctataacttcgag),

Ind23 (Fwd: catcattagagaatttacagaactacacttcattacaatgctccgta, Rev: tacggagcattgtaatgaagtgtagttctgtaaattctctaatgatg);

The constructs for Ind17 and Ind22 were synthesized (Twist Bioscience) as their mutations laid within the codon optimized regions and the resulting frameshift would differ from the original one. The splice-site mutations of Ind11, Ind15 [1], and Ind16 [2] were not included in the analysis.

### Sedimentation in continuous sucrose gradients

For sedimentation assays, cells were washed with PBS (Gibco) and lysed in cold 50 mM Hepes NaOH pH 7.5, 100 mM NaCl, 10 mM EGTA, 0.5% NP40, 1 mM DTT supplemented with 1X HALT Protease and Phosphatase Inhibitor Cocktail (Thermo Fisher Scientific). Lysates were homogenized by pushing them ten times through a 25G syringe and then the lysate was cleared by centrifugation for 10 min at 4 °C at 18,213 xg. 1 mg of total protein was loaded onto a 5% - 40% sucrose gradient which was prepared from lysis buffer supplemented with 5% or 40% sucrose (weight/volume) and mixed by the gradient master 108 (Biocomp). Samples were centrifuged in a SW60 rotor at 34,300 rpm for 16 hours at 4 °C, fractions collected from top and analyzed by immunoblotting.

### Immunoprecipitation and immunoblot analysis

K562 cells were washed with PBS and lysed in lysis buffer (LB) 50 mM Hepes NaOH pH 7.5, 100 mM NaCl, 10 mM EGTA, 0.5% NP40, 1 mM DTT supplemented with 1X HALT Protease and Phosphatase Inhibitor Cocktail (Thermo Fisher Scientific). Lysates were homogenized by aspirating and releasing it through 25G needles and cleared through centrifugation at 18,213 xg for 10 min at 4 °C. Monoclonal, anti-HA coupled agarose beads (Sigma, A2095-1mL) were used to immunoprecipitated HA-WDR26 from 1 mg total protein lysates over night at 4 °C on a rotator shaker. Precipitated proteins were washed extensively with LB, eluted in SDS sample buffer and analyzed by immunoblot.

To determine HBP1 binding to WDR26, HEK293 Flp-In Trex cells were transiently co-transfected with 2.5 µg of plasmid DNA encoding selected HA-tagged WDR26 variants and/or 2.5 µg of 3xFlag-tagged HBP1. HA-tagged WDR26 was captured with anti-HA-antibody coupled agarose beads (Sigma, A2095-1mL) for 2hrs at 4 °C on a rotator shaker. Precipitated proteins were eluted in SDS sample buffer and analyzed by immunoblot.

For immunoblot analysis, protein samples were blotted onto a 0.2 µm PVDF membrane (Amersham) and blocked in 3% milk in TBS-T if not indicated otherwise (10 mM Trizma pH 8.0, 150mM NaCl, 0.1% Tween 20). Membranes were incubated over night at 4 °C with the primary antibodies: HA (C29F4) Rabbit mAb (CST, 3724S, RRID: AB_1549585, lot 10+11, 1:2000), RANBP9 Rabbit pAB (Novus Biologicals, PAB16671, RRID: AB_10677213, lot 3, 1:1000), MAEA Sheep pAb (R&D Systems, AF7288, RRID: AB_10971438, CGG10119091, 1:1000), YPEL5 pAb (Thermo Fisher Scientific, PA5-26957, RRID: AB_2544457, lot VH3047907, 1:1000 in 5% BSA, TBS-T), ACTIN (CST, 4967L, RRID: AB_330288, lot 3, 1:1000) or DYKDDDDK Tag (D6W5B) Rabbit mAb (CST, 14793S, RRID: AB_2572291, lot 4, 1:1000). Membranes were washed with TBS-T and HRP-conjugated secondary antibodies Goat Anti-Rabbit, Goat Anti-Sheep and Goat-Anti-Mouse (Jackson ImmunoResearch, 1:10,000) were incubated for 1h at room temperature. To visualize bound antibodies, membranes were incubated with SuperSignal West PicoPLUS Substrate (Thermo Fisher Scientific) and imaged using the ChemiDoc Imaging System (BioRad) or ImageQuant 800 (GE Healthcare). Raw data of uncropped blots are provided in Fig. S1-S6.

### Structural analysis of SKDEAS-associated WDR26 mutants

To visualize the role of WDR26 as a supramolecular assembly module, we fit the AlphaFold2 (AF) [52] model of WDR26 dimer (lacking its disordered N-terminal region, Δ1-100), the AF model of the catalytic subunits (RMND5A-MAEA), and the prior cryo-EM structure of the scaffolding module RANBP9-TWA1-ARMC8 (extracted from PDB: 7NSC) into the cryo-EM map of the WDR26-CTLH complex (EMDB: EMD-12542).

To understand the impact of SKDEAS mutations on the YPEL5-binding capacity of WDR26, we first performed a focused refinement of a prior cryo-EM map of the CTLH subcomplex containing WDR26, RANBP9, TWA1, ARMC8, GID4, and YPEL5 (EMDB: EMD-12545) over the WDR26-YPEL5 region in Relion v4.0 [53, 54]. The generated map was deposited into Electron Microscopy Data Bank (EMDB) with the following identifier: EMD-19039. The position of YPEL5 within the WDR26-YPEL5 structure was refined by fitting its AF model into the corresponding part of the refined map in UCSF ChimeraX v1.5 [55]. For structure visualizations, we used PyMOL v2.5.2 (Schrödinger) or UCSF ChimeraX v1.5 [55].

## Results

### SKDEAS-associated WDR26 mutations and their structural prediction on altered CTLH E3 complex assembly

Recent mechanistic studies have provided cryo-EM maps for various WDR26-containing assemblies [11, 21]. To gain insights into the structural roles of WDR26 in these complexes, we first obtained an AlphaFold2 (hereafter abbreviated AF [52]) models of WDR26 dimer and the catalytic (RMND5A-MAEA) module, which we fitted, along with the prior structure of the scaffolding subunits (RANBP9-TWA1-ARMC8), into the published 19 Å-resolution cryo-EM map of the WDR26-CTLH E3 assembly (Fig. 1A). These data suggest that WDR26 connects the core-CTLH E3 complexes into a singular oval-shaped supramolecular assembly, reminiscent of yeast Gid7 driving formation of the Chelator-GID E3 [10, 11, 21] (Fig. 1B).

In the context of the WDR26-CTLH E3, the multiple domains of WDR26 play two structural functions (Fig. 1C). First, the LisH domains and the C-terminal parts of the CRA domains (referred to as CRA^C^) from two WDR26 protomers promote formation of the WDR26 homodimer. This dimerization interface is further buttressed by the edges of the WD40 β-propellers and short C-terminal helices binding *in trans* to the opposite WDR26 protomer. Second, the helical units formed by the CTLH domains and the N-terminal part of the CRA domain (referred to as CRA^N^) project outward from each WDR26 protomer to contact the analogous CTLH-CRA^N^ region from the neighboring scaffolding subunit RANBP9. Meanwhile, another scaffolding subunit TWA1 associates with RANBP9 to connect the WDR26 dimer to the E3 ligase module, thereby completing the arrangement of CTLH modules (Fig. 1A).

We set out to analyze pathogenic *de novo* variants of the *WDR26* gene from currently 26 published cases with detailed clinical histories [1–6, 8]. In addition to five splice-site mutations, these *WDR26* variants include seven missense, nine frameshift, and eight nonsense mutations. Prior studies mapping these latter mutations onto the primary sequence of WDR26 showed that they occur in different regions rather than clustering to a specific domain [1–6, 8] (Table S1). Except for four short truncations that do not contain any known WDR26 functional regions, the pathogenic variants of 16 individuals (hereafter, “Ind”) can be categorized, based on the domain in which a given point mutation occurs or the position from which a truncation or a frameshift originates, as LisH-CTLH-CRA mutations (Leu215Pro (Ind4), Gln302Aspfs*22 (Ind5), Asp284Asn (Ind6), Ser254Arg (Ind9), Arg279* (Ind12), Ile192Asnfs*8 (Ind13), and Trp172Arg (Ind14)), and WD40 β-propeller mutations (Glu426* (Ind1), His389Profs*6 (Ind2), Val486Glufs*9 (Ind3), Gln524* (Ind8), Trp428* (Ind10), Gln348* (Ind19), Val619Glufs*16 (Ind20), Arg510Gln (Ind21), and Trp359* (Ind23)) (Fig. 1C and 1D).

**Fig. 2:**
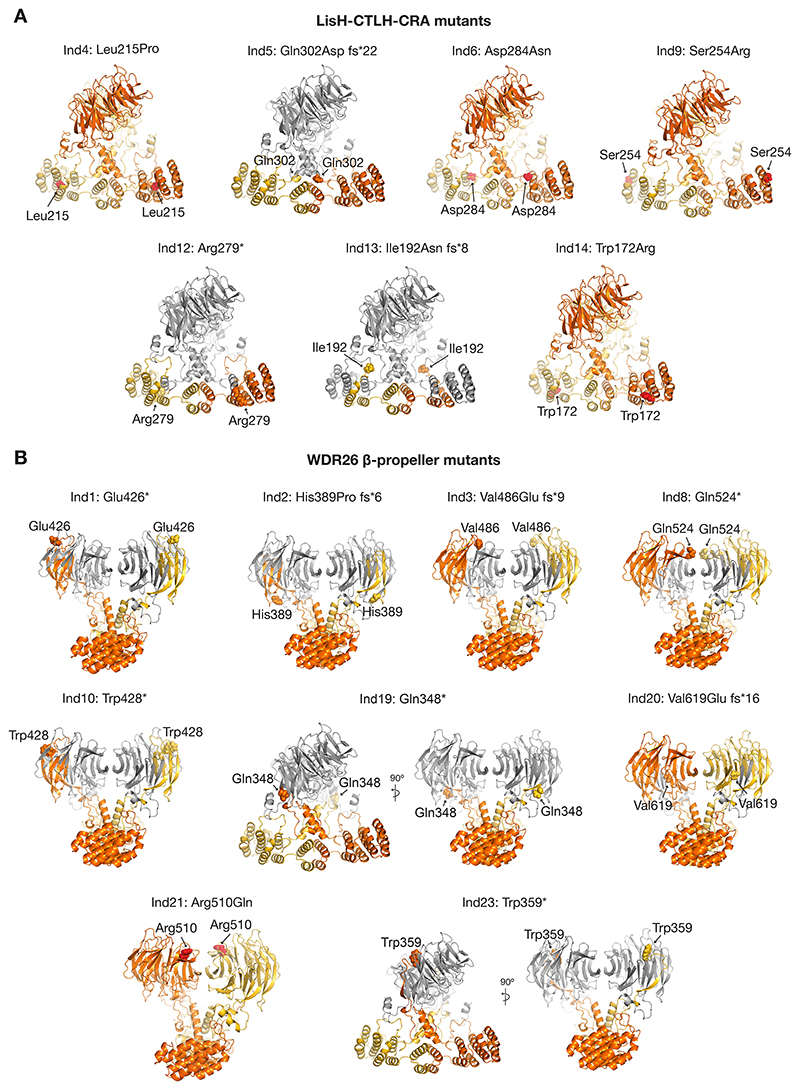
AlphaFold2-based structural prediction of WDR26 SKDEAS mutants. A) AF model of WDR26 dimer indicating the structural location of the SKDEAS-associated mutations within the LisH-CTLH-CRA domain. Single amino acid substitutions are indicated with red spheres, whereas the truncated parts are shown in grey. B) Similar to A) but highlighting the locations of the WDR26 β-propeller mutants.

The intricate web of domain interactions within the WDR26 dimer combined with its crucial role in mediating the CTLH E3 supramolecular assembly raise the possibility that SKDEAS-associated mutants could be detrimental for the formation of functional WDR26-CTLH E3 complexes. Indeed, mapping the pathogenic mutations to the AF model of the WDR26 dimer showed that many of them cause truncations within the LisH-CRA^C^ as well as the CTLH-CRA^N^ units, and/or partial or complete elimination of the β-propeller domain (Fig. 2A and 2B). In addition, numerous mutations result in single amino acid replacements within the functionally relevant WDR26 domains, potentially interfering with WDR26’s structural functions (Fig. 2A and 2B). Taken together, both categories of mutants likely affect WDR26 homodimerization interface and/or its interactions with other CTLH subunits, thereby disrupting the WDR26-CTLH E3 supramolecular assembly.

### WDR26 mutants affect formation of supramolecular WDR26-CTLH assembly

**Fig. 3:**
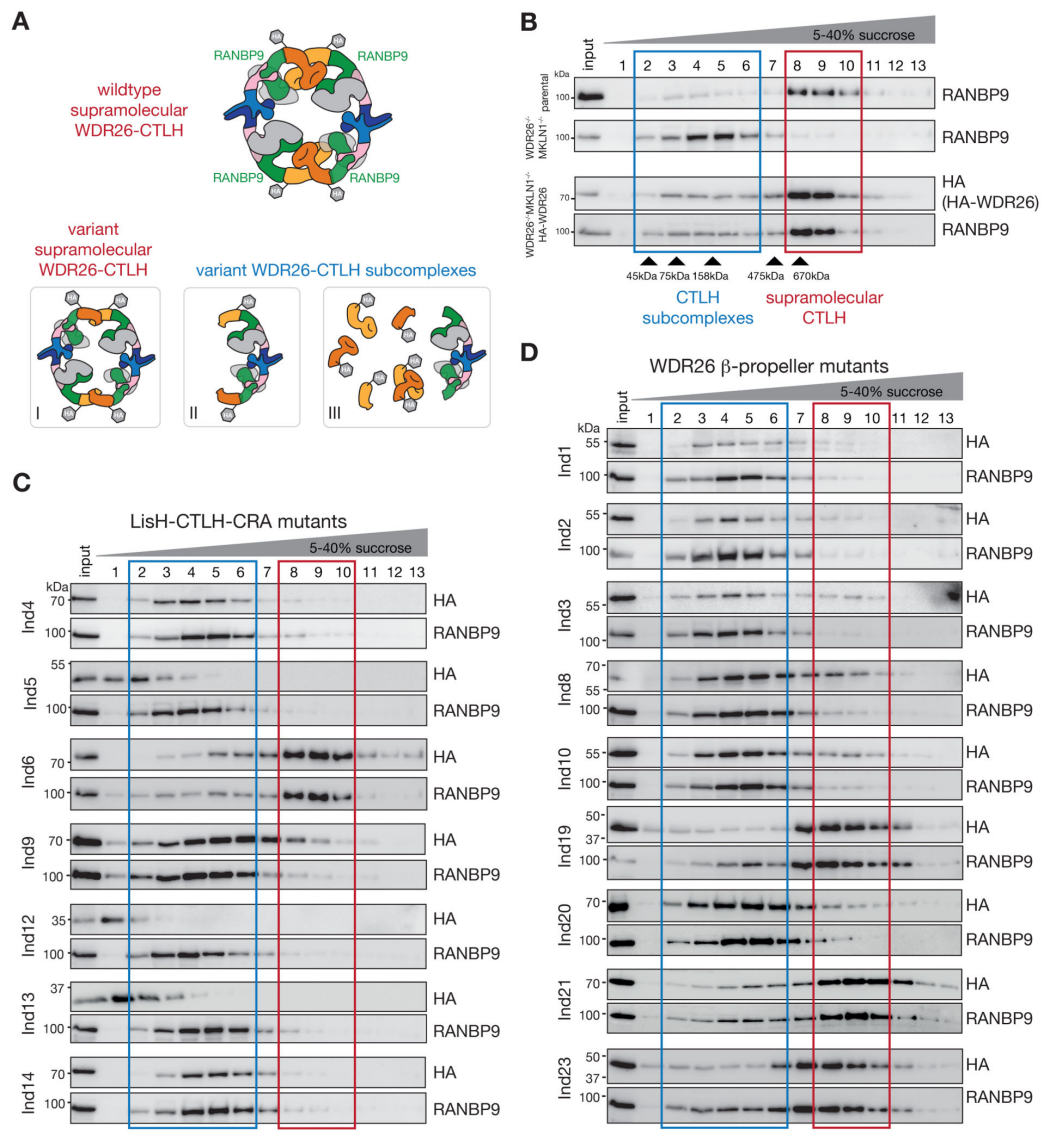
WDR26 mutants affect formation of supramolecular WDR26-CTLH E3 assembly. A) Cartoon of the wildtype supramolecular CTLH E3 assembly and the potential CTLH (sub-) complexes (I, II, and III) caused by WDR26 SKDEAS-associated mutations. Group I: variants maintaining formation of the supramolecular CTLH E3 assembly; group II: variants disrupting CTLH E3 supramolecular assembly but retaining interactions with RANBP9; and group III: variants abolishing both the higher-order CTLH E3 assembly and interactions with RANBP9. HA-tagged WDR26 subunits are indicated. B) Cell lysates of K562 parental, *WDR26-* and *MKLN1*-deficient double knockout K562 cells (*WDR26*^*-/-*^; *MKLN1*^*-/-*^), and *WDR26*^*-/-*^; *MKLN1*^*-/-*^ cells with stably reintroduced HA-tagged WDR26 were fractionated on a continuous 5-40% sucrose gradient, and fractions analyzed by immunoblotting. Fractions with supramolecular assemblies >670 kDa are indicated with a red box, and smaller subcomplexes with a blue box. C)-D) Cell lysates of K562 *WDR26*^*-/-*^; *MKLN1*^*-/-*^ stably reintroduced HA-tagged WDR26 variants from different individuals (Ind#) were fractionated on a continuous 5-40% sucrose gradient, and fractions analyzed by immunoblotting. Supramolecular assemblies and subcomplexes are boxed in red and blue, respectively. Immunoblot data of individual WDR26 variant are grouped into LisH-CTLH-CRA mutants C) and WDR26 β-propeller mutants D). Uncropped blots are provided in Fig. S1-S3.

To monitor the capacity of WDR26 mutations for mediating supramolecular assemblies of CTLH E3, we established a system for expressing each mutant individually as the only cellular Gid7 ortholog (Fig. 3A). We made use of our K562 knockout cell line where genes encoding both human Gid7 family members *WDR26* and *MKLN1* were deleted [11]. In contrast to parental control K562 cells, lysate of *WDR26*^*-/-*^; *MKLN1*^*-/-*^ double knockout cells fractionated on a continuous sucrose gradient did not show the CTLH scaffold subunit RANBP9 detectably migrating in the high molecular weight fractions (fraction eight to ten) previously shown to correspond to the supramolecular CTLH complexes (Fig. 3A, 3B, and S7A) [11]. The stable expression of HA-tagged WDR26 in *WDR26*^*-/-*^; *MKLN1*^*-/-*^ cells triggered the reassembly of WDR26-mediated supramolecular complexes, as indicated by the co-sedimentation of HA-WDR26 and the CTLH core subunit RANBP9 in fractions eight to ten (Fig. 3B and S7B).

Next, we generated *WDR26*^*-/-*^; *MKLN1*^*-/-*^ cells expressing SKDEAS WDR26 mutants. For 16 mutants, for which we could detect expression of the re-introduced HA-tagged WDR26, we monitored their sedimentation and that of RANBP9 in continuous sucrose gradients. Twelve WDR26 mutants failed to form the supramolecular CTLH E3 assembly (Fig. 3C and 3D), including the majority of mutants in the LisH-CTLH-CRA domain (with exception of Ind6) (Fig. 3C). All six WDR26 mutants with partial truncations of the WD40 β-propeller domain also impaired formation of high molecular weight complexes (Fig. 3D). By contrast, the WDR26 mutations of Ind19 and Ind23, both with a complete elimination of all β-propeller blades (i.e. lacking capacity to form even a partial sheet) still formed high molecular weight complexes and co-sedimented with RANBP9. So did two full-length WDR26 variants (Ind6 and Ind21) with conservative mutations that would seem to maintain the overall structure. Indeed, the Asp284Asn substitution (Ind6) maps to the LisH-CRA^C^/CTLH-CRA^N^ interface, where it could largely maintain interactions with neighboring residues (Fig. 2A and 3C). Similarly, the Arg510Gln residue substitution in Ind21 is conservative and maps to the surface of the β-propeller domain (Fig. 2B and 3D). Taken together, the data highlight the elaborate nature of interactions between the elements mediating WDR26 self-assembly.

### WDR26 mutants exhibit binding defects with core-CTLH E3 complex

Loss of the WDR26-dependent shift of the scaffolding subunit RANBP9 in sucrose gradients towards the high molecular weight fractions indicates lack of the supramolecular CTLH E3 complex formation. In addition, for four of the WDR26 mutants with impaired capacity for mediating higher order CTLH assembly (Ind5, Ind9, Ind12, and Ind13), the sedimentation profiles in sucrose gradients differed from that of RANBP9, indicating these mutants do not associate with RANBP9-containing CTLH subcomplexes (see subcomplex types of group III, Fig. 3A). This is rationalized by mutational effects on the structure: Ind12 and Ind13 delete substantial portions of the CTLH-CRA^N^ helical unit, which binds RANBP9 (Fig. 2A). The same domain is affected in Ind9, which carries a single residue substitution (Ser254Arg) at the external face of the CTLH-CRA^N^ domain right at its interface with RANBP9. The corresponding sedimentation profile of HA-WDR26 towards higher molecular weight fractions might present WDR26 oligomers as recently described [31]. Meanwhile, Ind5 deletes the entire CRA^C^ domain, which contributes to WDR26 homodimerization, and is presumably required for proper folding of the CTLH-CRA-LisH region (Fig. 2A).

**Fig. 4:**
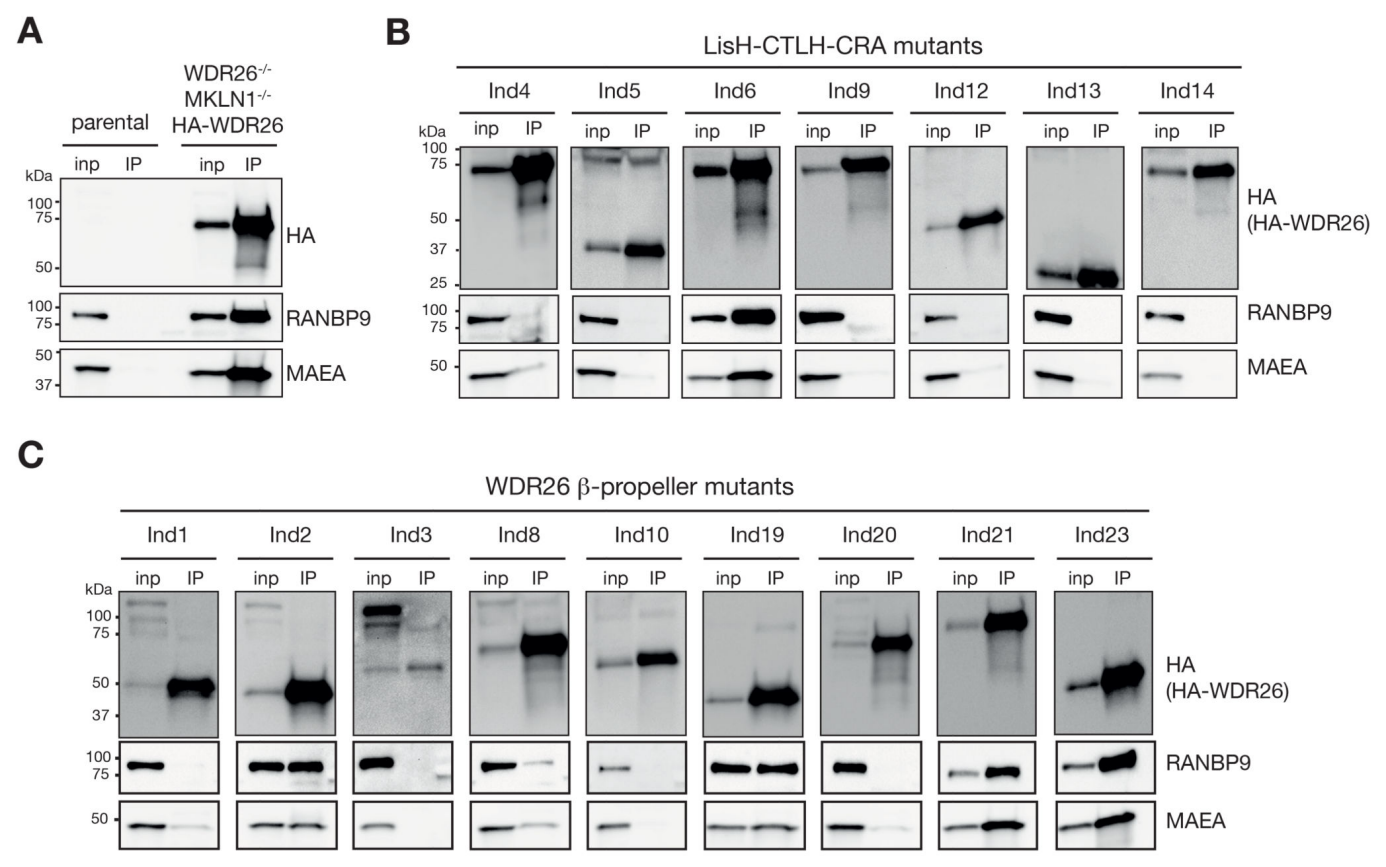
WDR26 mutants exhibit binding defects with core-CTLH E3 complex. A) HA immunoprecipitation from *WDR26*^*-/-*^; *MKLN1*^*-/-*^ cells stably expressing HA-tagged WDR26 and parental control cells. Input (inp) and immunoprecipitants (IP) were analyzed by immunoblotting. B)-C) HA immunoprecipitation from *WDR26*^*-/-*^; *MKLN1*^*-/-*^ cells stably expressing HA-tagged WDR26 variants from different individuals (Ind#). Immunoblot analysis of inp and IP-captured proteins for each individual are presented separately for efficient detection (expression levels largely vary between WDR26 variants). Uncropped blots are provided in Fig. S4.

The contents of complexes in lower molecular weight fractions is less clear for several other WDR26 mutants, because they could potentially form assorted subcomplexes, or mixtures of subcomplexes, of molecular weights similar to those formed by RANBP9. Thus, to further characterize the potential of WDR26 mutants to bind other CTLH subunits, we performed immunoprecipitations of HA-WDR26 mutants expressed in *WDR26*^*-/*^,^*-*^ *MKLN1*^*-/-*^ cells. Wildtype HA-WDR26 efficiently co-precipitated subunits from both, the scaffold and catalytic modules (represented by RANBP9 and MAEA, respectively) of the core-CTLH (Fig. 4A). Applying the same assay platform for WDR26 mutants confirmed both that the four variants (Ind6, Ind19, Ind21, and Ind23) found in high molecular weight assemblies retain interactions with CTLH subunits, and that the Ind5, Ind9, Ind12, and Ind13 mutations indeed hinder CTLH E3 complex formation (Fig. 4B and 4C). Furthermore, mutations of Ind1, Ind3, Ind8, Ind10, and Ind20 variants, which all produce at least one β-propeller blade, likewise vastly impaired interaction with the core-CTLH modules. This can be explained in case the extensive hydrophobic surfaces exposed in the blades of a partial WDR26 β-propeller would impair its folding. Along the same lines, Ind2, which would produce a protein without a single blade of the β-propeller, showed substantial binding to RANBP9 and MAEA, a property shared with other mutants that maintain the LisH-CTLH-CRA domain (Fig. 4C). Finally, single residue substitutions within the center of the CTLH-CRA^N^ domain also resulted in a binding defect. Notably, both the Leu215Pro mutation in Ind4, which replaces a helix-favoring residue (Leu) with the most helix-destabilizing residue (Pro), and the Trp172Arg in Ind14 could destabilize and/or remodel this RANBP9-binding domain (Fig. 2A).

### WDR26 mutations affect expression of and/or association with YPEL5

**Fig. 5:**
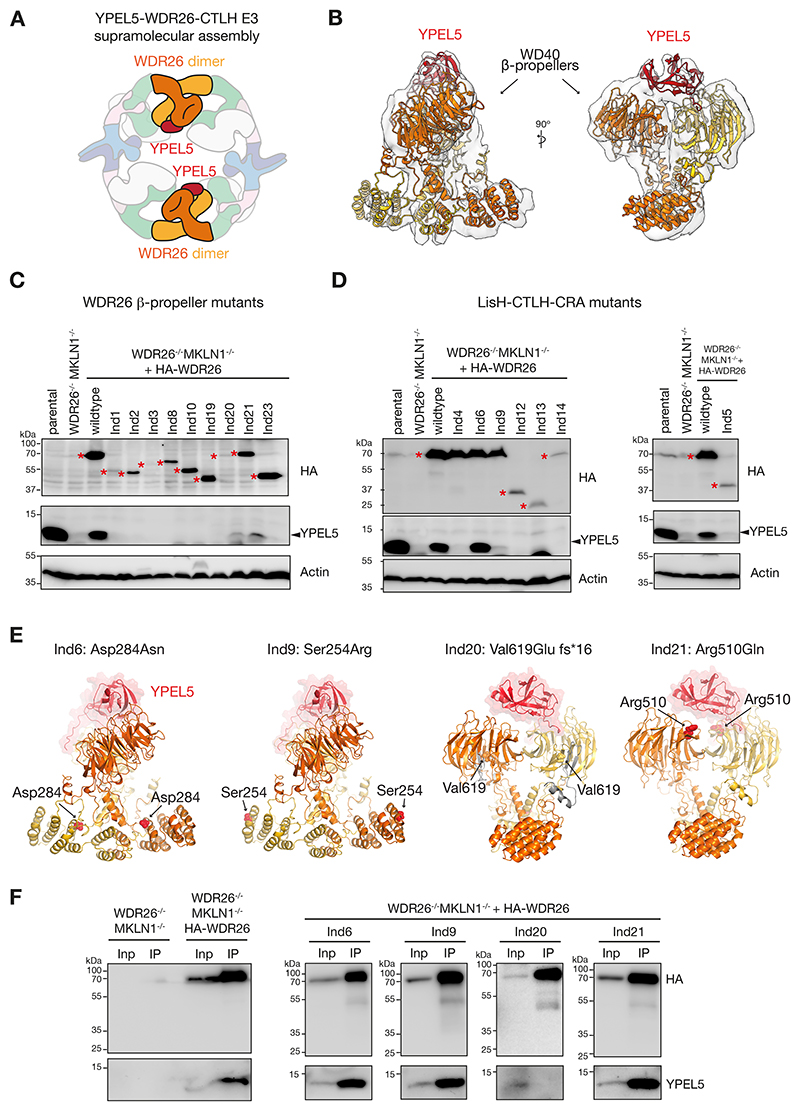
WDR26 mutations affecting association with YPEL5. A) Cartoon of WDR26-CTLH E3 highlighting the mode of YPEL5 interactions with WDR26 dimers. CTLH subunits are color-coded as in Figure 1A, whereas YPEL5 is colored red. B) 6.7-Å-resolution map obtained by focused refinement of the prior cryo-EM reconstruction of the WDR26-containing sub-complex (EMD-12545). The resulting map was fitted with the AF model of WDR26 dimer in complex with YPEL5, whose position with respect to the WDR26 dimer was refined by fitting in the electron density. C) and D) Whole cell lysates of K562 parental, *WDR26*^*-/-*^; *MKLN1*^*-/-*^, and *WDR26*^*-/-*^; *MKLN1*^*-/-*^ cells stably expressing HA-tagged wildtype or variant WDR26 were analyzed by immunoblotting (indicated by red stars). Actin serves as loading control. E) AF model of YPEL5-WDR26 dimer complex indicating the structural location of the SKDEAS variants showing impaired YPEL5 levels as indicated in C) and D). Single amino acid substitutions are indicated with red spheres, whereas truncated parts shown in grey. F) HA-tagged wildtype or WDR26 variants from different individuals (Ind#) were stably expressed in *WDR26*^*-/-*^; *MKLN1*^*-/-*^ cells and captured by HA-immunoprecipitation (IP). Immunoblot analysis of input (Inp) and IP-captured proteins (IP) for each individual are presented separately for efficient detection (expression levels largely vary between WDR26 variants). Uncropped blots are provided in Fig. S5.

Four WDR26 variants, Ind6, Ind19, Ind21, and Ind23, neither showed defects in supramolecular assembly, nor binding to the CTLH subunits from the two core modules (Table 1). Of these, Ind19 and Ind23 lack the WD40 β-propeller, whereas Ind21 has a single missense mutation in this domain. The only role for the WDR26 β-propeller, that has been structurally characterized to date, is binding to the CTLH E3-associated protein YPEL5 [11] (Fig. 5A). To explain the effect of SKDEAS-associated WDR26 mutants on their YPEL5-binding capacity, we performed a focused refinement of the previous map of WDR26-CTLH subcomplex over the YPEL5-bound WDR26 dimer (Fig. 5B). Fitting the resulting 6.7-Å-map with an AF model showed one molecule of YPEL5 asymmetrically interacting with both WDR26 β-propellers, suggesting that their truncation or disruption of WDR26 homodimerization could impair YPEL5 binding.

As a first step toward investigating whether the association of YPEL5 with WDR26 was altered by the mutants, we monitored endogenous YPEL5 amounts by immunoblot analysis. YPEL5 was barely detectable in lysates of *WDR26*^*-/-*^; *MKLN1*^*-/-*^ cells, but restored after expression of HA-tagged wildtype WDR26 (Fig. 5C). Thus, in this assay system, YPEL5 amounts are dependent on WDR26 and therefore a readout for functional WDR26. WDR26 binding presumably prevents exposure of an extensive hydrophobic surface or degron to stabilize YPEL5. Only *WDR26*^*-/-*^; *MKLN1*^*-/-*^ cells expressing the mutant of Ind6 showed significant YPEL5 expression to levels observed in wildtype cells (Fig. 5C and 5D). Expression of WDR26 mutant of Ind9, Ind20, and Ind21 weakly restored YPEL5 amounts, whereas no other tested WDR26 mutants showed substantial rescue (Fig. 5C and 5D). In particular, despite retaining other functionality, neither the Ind19 nor Ind23 WDR26 mutant rescues YPEL5 protein level, consistent with their complete lack of YPEL5-binding β-propeller domain. Finally, AF model showed that the single amino acid substitutions in Ind6, Ind9, and Ind21 maintain the β-propeller domain (Fig. 5E), consistent with immunoprecipitation analysis further indicating that these WDR26 mutants could co-precipitate YPEL5 (Fig. 5F). However, mutation of Ind20 with a partial truncation of the β-propeller eliminates interaction with YPEL5, despite rescuing low YPEL5 expression levels.

### WDR26 mutations attenuate CTLH E3 binding to HBP1

**Fig. 6:**
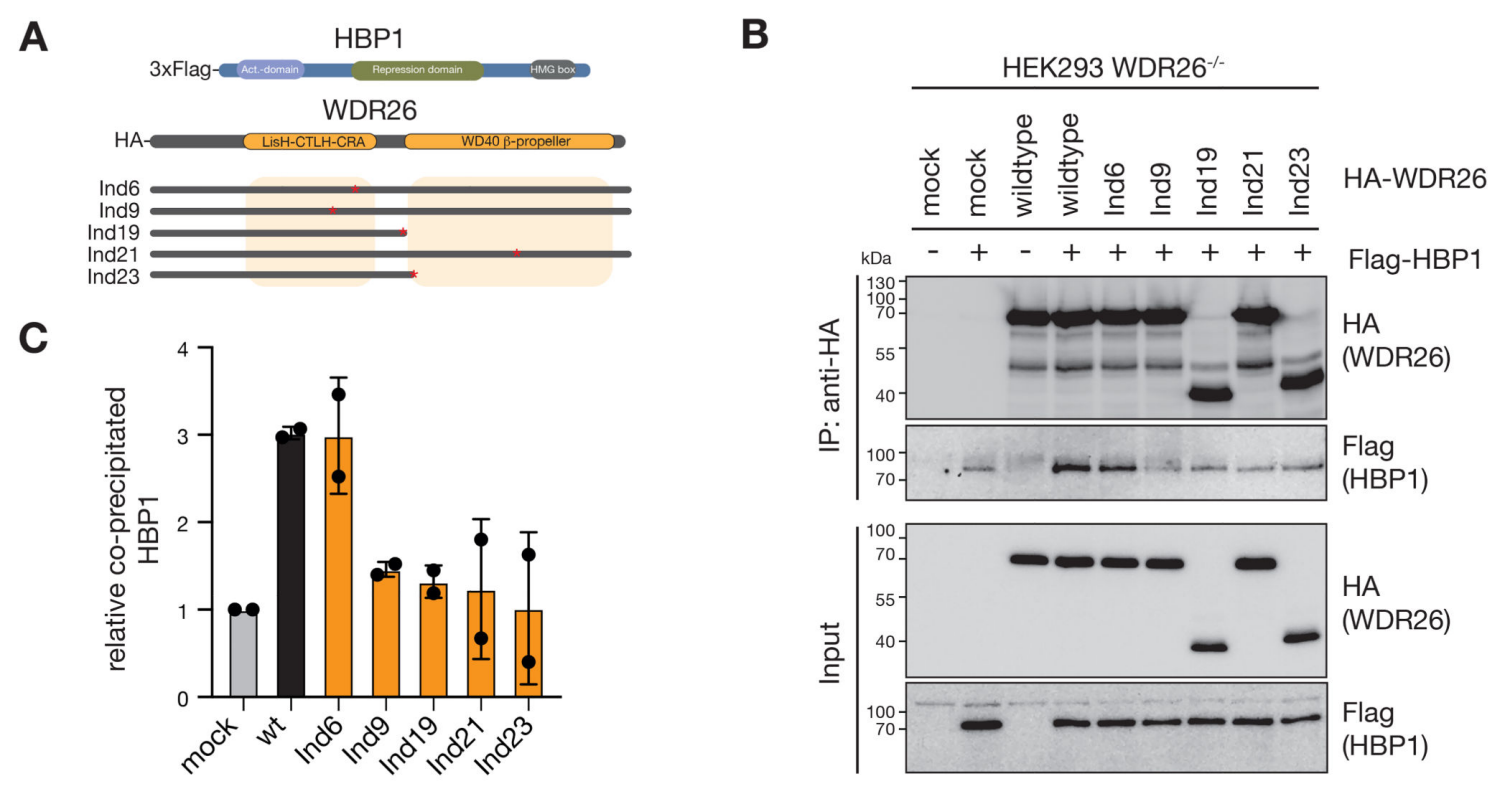
Attenuated HBP1 binding by WDR26 mutations. A) Cartoon representing HA-tagged WDR26 variants tested for 3xFlag-HBP1 association. B) HEK293 *WDR26*^*-/-*^ knockout cells were mock or co-transfected with 3xFlag-tagged HBP1 and/or HA-tagged WDR26 variants as indicated. HA-WDR26 was captured from cell lysates by HA-immunoprecipitation and precipitated proteins were analyzed by immunoblotting. C) Quantitation of Flag-HBP1 immunoblot signals from B) normalized to 3xFlag-HBP1 input and relative to mock-IP value (in B lane 2). Graph shows results by means +/- SD of n=2. Uncropped blots are provided in Fig. S6.

The other CTLH complex binding partner known to be dependent on WDR26 - and probably its β-propeller domain - is the HMG-box transcriptional repressor HBP1 [21, 29]. We established an assay examining this interaction upon co-transfection of HEK293 cells deficient for WDR26 (*WDR26*^*-/-*^) with HA-tagged WDR26 and/or 3xFlag-tagged HBP1 (Fig. 6A). Wildtype HA-WDR26 co-immunoprecipitated 3xFlag-HBP1 indicating robust interaction between WDR26 and HBP1 (Fig. 6B). We next tested effects of five selected WDR26 variants: four that retain capacity to form high molecular weight CTLH complexes (Ind6, Ind19, Ind21, and Ind23), and Ind9. The latter one showed lost interaction with the scaffolding and E3 ligase module subunits, limited rescue of YPEL5 expression, but despite this still could bind YPEL5 (Table 1). All WDR26 mutants, except Ind6, fail to co-precipitate HBP1 above background levels (Fig. 6B and 6C). In summary, WDR26 variants impair binding to known interaction partners, however, not necessarily in the same way, suggesting that mutations might affect distinct binding properties.

## Discussion

For sixteen tested SKDEAS-associated WDR26 mutations, we found that fifteen showed defects in known WDR26-CTLH complex functions (Table 1) [11, 21, 29]. The majority of these mutants were defective in the formation of supramolecular complexes, association with scaffolding RANBP9 and E3 ligase MAEA subunits, rescuing expression of and binding to YPEL5, and binding to HBP1. Five WDR26 mutants lost some CTLH E3 functions but not others. For example, Ind2, bearing a truncation at the first blade of the WDR26 β-propeller, maintained ability to bind CTLH subunits, but lost the ability to mediate supramolecular WDR26-CTLH assembly, showing that these are separable functions (see subcomplex type of group II, Fig. 3A). The Ind2 mutant also failed to rescue YPEL5 expression in accordance with its lack of the majority of the β-propeller domain, and also lost binding to HBP1, presumably due to β-propeller defects. Of the three mutants (Ind6, Ind9, and Ind21) that did bind YPEL5, Ind9 (Ser254Arg in the LisH-CTLH-CRA domain) and Ind21 (Arg510Gln in the fourth β-propeller blade) lost binding to HBP1, consistent with substrate binding being a crucial feature of WDR26 function that extends to both of the main domains of WDR26.

WDR26 is a multidomain protein containing the LisH-CTLH-CRA motif that mediates dimerization and binding to the scaffold subunit RANBP9, and also the WD40-type β-propeller domain. A priori, one might expect that there would be common effects of mutations in a given domain. Indeed, an intact β-propeller was a requirement for binding to YPEL5 and HBP1. However, we found that this was not sufficient, as exemplified by Ind21, which as noted above retains binding to YPEL5, but not to HBP1 under our experimental conditions. Moreover, the β-propeller of WDR26 is not obligatorily required for binding CTLH subunits and higher order assembly. As shown by WDR26 mutants of Ind19 and Ind23, which bear complete or near-complete truncations of the β-propeller domain respectively, supramolecular assembly complexes with RANBP9 are formed. However, the Ind2 truncation, which just retained the first blade of the WDR26 β-propeller, binds RANBP9 but lost the ability to form supramolecular assemblies. Hence, the precise location of a truncation in the β-propeller domain clearly matters, as truncation mutants with slightly more or fewer amino acids differentially effect binding to CTLH subunits and/or supramolecular assembly. Further work using saturation mutagenesis and structural studies using SKDEAS WDR26 mutant forms will be necessary to tease apart exactly how WDR26 controls both supramolecular complex formation and substrate binding. Finally, we note that Ind9, containing a single point mutation (Ser254Arg) in the LisH-CTLH-CRA domains, lost binding to HBP1. We speculate that subtle changes in LisH-CTLH-CRA domain could affect the orientation or dynamics of the adjacent β-propellers within the WDR26 dimer, or other intricate features of assembly that ultimately regulate capture of substrates, such as HBP1.

WDR26-CTLH E3 complexes are amongst a cohort of multiprotein E3 ligases mutated in diseases. It thus may be instructive to consider disease-causing mutations that effect protein binding and assembly properties, in well characterized subunits of other E3 ligase complexes. A notable paradigm is the tumor suppressor SPOP, which recruits GLI2, BRD2-4, and numerous other crucial cellular regulators to a core scaffold/catalytic E3 ligase module (CUL3-RBX1) for ubiquitin-mediated degradation [56, 57]. SPOP is a multidomain protein, with a substrate binding domain and two dimerization domains one of which also binds CUL3 [58]. Mutations across various SPOP domains cause different cancers [56, 57]. Some mutations map to the substrate-binding site and clearly impair binding to all known substrates. However, the roles of other mutations differentially impact the stability of various substrates. Potential mechanistic effects have only emerged recently, with the discovery that SPOP forms self-assembled filaments, and the relative arrangements of domains in the self-assemblies may differ for different cancer mutants [59, 60]. The different filaments show distinct stabilities, and presumably also control recruitment of particular substrates although this latter functionality remains to be clearly elucidated. With this in mind, some SKDEAS WDR26 mutants not only disrupt WDR26 dimerization (Ind5, Ind12, and Ind13), but might trigger formation and stabilization of higher-order WDR26 oligomers in case core-CTLH E3 binding is impaired [31]. Indeed, mutants of Ind8 and Ind9 sediment in high molecular weight fractions distinct to the RANBP9 sedimentation profile, indicative for WDR26 self-assemblies. Moreover, we noted that one mutation, Ind6 (Asp284Asn) with a single, conservative point mutation manifests similar biochemical properties to the wildtype WDR26 in our assay platforms. We speculate that the Ind6 mutation may either be representative of a cell-type specific alteration in CTLH functionality, or subtly affect self-assembly in such a way as to impact ubiquitylation of some substrates but not others. Notably, in Ind6 additional mutations in loci *ARID1B* and *ZMYND11* have been described which might account, at least in part, for SKDEAS [1], of which *ZMYND11* was recently associated with disability disorders [61].

A key question for the future is the number and nature of different supramolecular CTLH E3 assemblies. At this point it remains unclear whether WDR26 - or the other Gid7 ortholog MKLN1 - form exclusive, or mixed supramolecular assemblies, and whether they have overlapping functions or can compensate for each other. Despite their common LisH, CTLH, and CRA domains, they differ in other functionally important domains [11, 31, 32]. WDR26 has a WD40 repeat β-propeller, which dictates binding specificity for YPEL5 and presumably substrates such as HBP1 [11, 21, 29]. In contrast, the MKLN1 β-propeller is a different structure and in addition, MKLN1 contains a Discoidin domain. Moreover, MKLN1 forms a tetramer or even higher molecular weight self-assembly rather than a dimer [31, 32], and is thought to bind a unique set of substrates [28]. Thus, despite the common ability of WDR26 and MKLN1 to form higher-order assemblies, there is presently no evidence that they display homologous surfaces to recruit substrates. More work will be needed to determine the substrates and structural mechanisms mediating their targeting. This will be particularly relevant for SKDEAS WDR26 mutations, given their polymorphic nature encompassing truncation mutations and single point mutations in the WDR26 coding region.

A reasonable assumption from the overlapping common phenotypes of SKDEAS is that WDR26 affects neurons and/or other cells in the nervous system [41]; as our data show, when mutated, WDR26 affects one or more CTLH functions. Given the diversity of WDR26 mutations, it seems likely that WDR26 CTLH E3 complexes control one or more vital function(s) in nervous system development that is ultimately manifested as SKDEAS. One plausible WDR26 function could be the CTLH E3-dependent elimination of a key substrate required for neuronal development as suggested for the epigenetic factor TET2 [41]. Importantly, very few CTLH E3 substrates have been validated so far, raising the possibility that new CTLH functions can be uncovered in neurons or other neuron-associated cells. As new technologies are developed that will enable identifying E3 ligase substrates in very small numbers of cells, such functions will likely emerge from analyzing SKDEAS patient samples.

## Supplementary Material

Supplemental material

## Figures and Tables

**Table 1:** Summary of biochemical assays. + (weak) to +++ (strong), - (lack), NT (not tested)

WDR26 variant	Form supra-molecular complex	Binding core CTLH E3	Rescue YPEL5 expression	Binding YPEL5	Binding HBP1
**WT**	**+++**	**+++**	**+++**	**+++**	**+++**
**Ind1**	**-**	**+**	**-**	NT	NT
**Ind2**	**-**	**++**	**-**	NT	NT
**Ind3**	**-**	**-**	**-**	NT	NT
**Ind4**	**-**	**-**	**-**	NT	NT
**Ind5**	**-**	**-**	**-**	NT	NT
**Ind6**	**+++**	**+++**	**+++**	**+++**	**++**
**Ind8**	**-**	**+**	**-**	NT	NT
**Ind9**	**-**	**-**	**+**	**+++**	**-**
**Ind10**	**-**	**-**	**-**	NT	NT
**Ind12**	**-**	**-**	**-**	NT	NT
**Ind13**	**-**	**-**	**-**	NT	NT
**Ind14**	**-**	**-**	**-**	NT	NT
**Ind19**	**+++**	**++**	**-**	NT	**-**
**Ind20**	**-**	**+**	**+**	**-**	NT
**Ind21**	**+++**	**+++**	**++**	**+++**	**-**
**Ind23**	**+++**	**+++**	**-**	NT	**-**

## Data Availability

All relevant data are provided in the main article and in the supplement file. The generated cryo-EM map was deposited into Electron Microscopy Data Bank (EMDB) with the following identifier: EMD-19039.
