# Supplementary material for: Skraban-Deardorff intellectual disability syndrome-associated mutations in WDR26 impair CTLH E3 complex assembly
Source: FEBS Lett. Author manuscript; Available in PMC 2024 Oct 16. (PMC7616460; doi:10.1002/1873-3468.14866)
Supplement: Supplemental material [file EMS198546-supplement-Supplemental_material.pdf]

Skraban-Deardorff intellectual disability syndrome-associated mutations in WDR26 impair CTLH E3 complex assembly

Annette Gross[1,2], Judith Müller[2], Jakub Chrustowicz[2], Alexander Strasser[1], Karthik V. Gottemukkala[2], Dawafuti Sherpa[2], Brenda A. Schulman[2], Peter J. Murray[1,#] and Arno F. Alpi[2,#]

[1] Immunoregulation Research Group, Max Planck Institute of Biochemistry, Martinsried, Germany.

[2] Department of Molecular Machines and Signaling, Max Planck Institute of Biochemistry, Martinsried, Germany.

[#]Corresponding authors: murray@biochem.mpg.de and aalpi@biochem.mpg.de

Supporting information

Fig. S1. Original scans of Fig. 3B.

Fig. S2. Original scans of Fig. 3C.

Fig. S3. Original scans of Fig. 3D.

Fig. S4. Original scans of Fig. 4A-D.

Fig. S5. Original scans of Fig. 5C, 5D, and 5F.

Fig. S6. Original scans of Fig. 6B.

Fig. S7. Analysis of CTLH E3 complex assembly using sucrose density gradient centrifugation.

Table S1. List of *de novo* WDR26 mutations from 26 published cases with clinical history.

Figure 3B

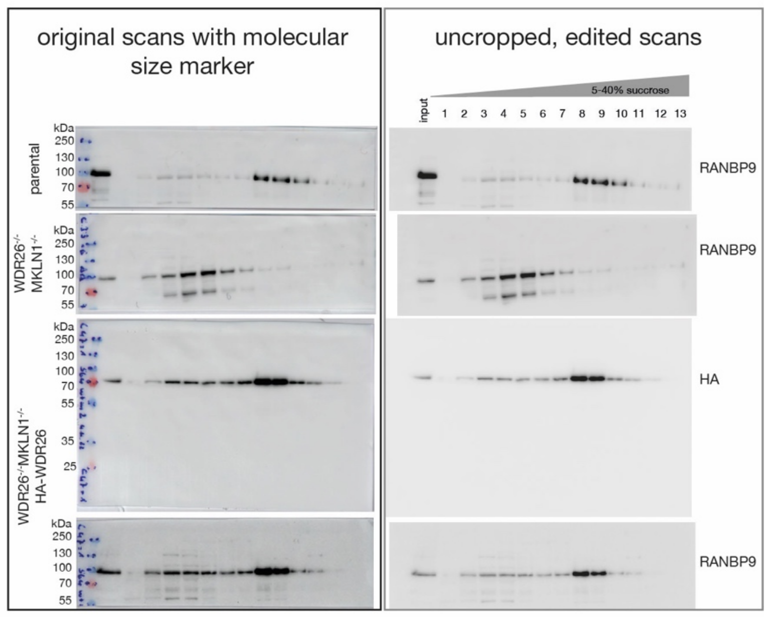

Fig. S1. Original scans of Figure 3B.

Figure 3C

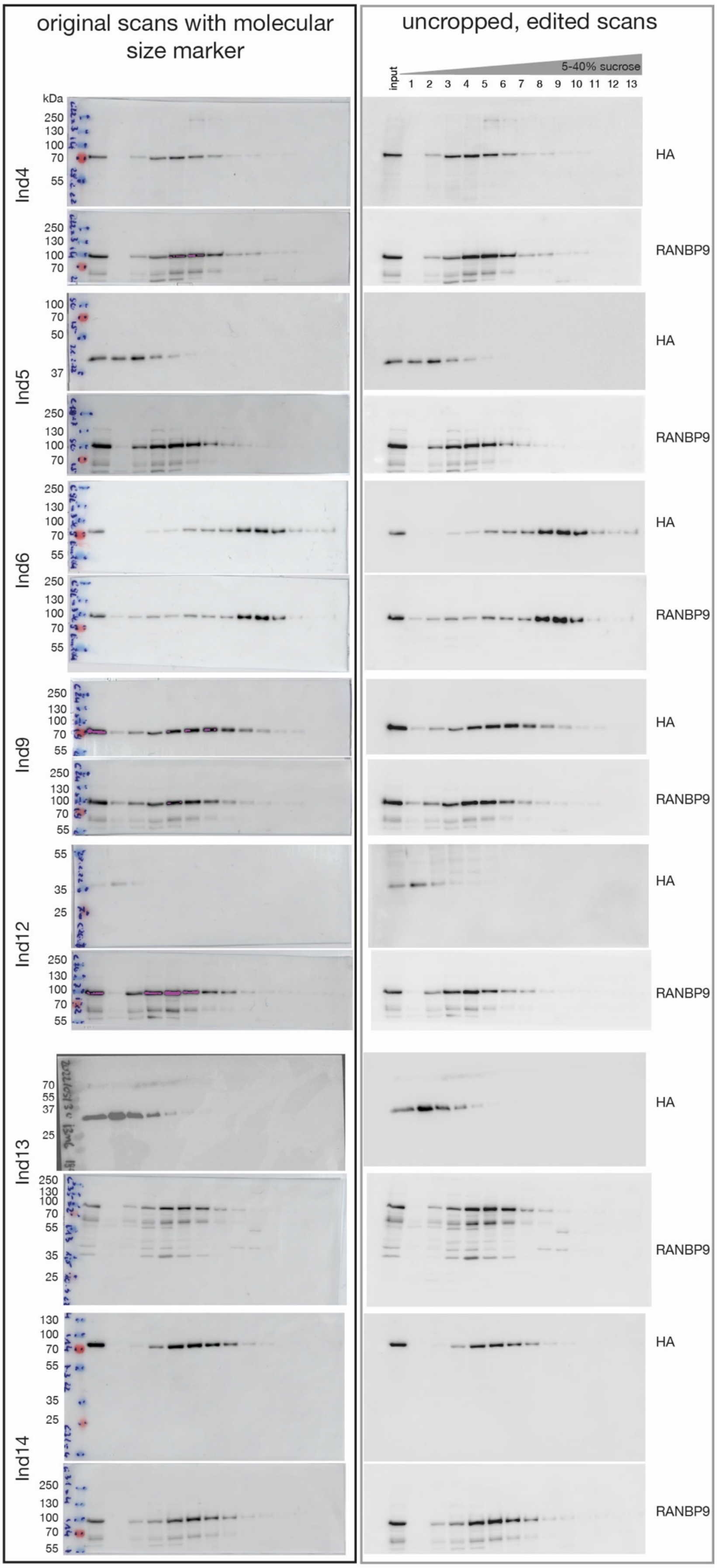
original scans with molecular size marker
uncropped, edited scans
input
1 2 3 4 5 6 7 8 9 10 11 12 13
5-40% sucrose
kDa
Ind4
Ind5
Ind6
Ind9
Ind12
Ind13
Ind14
HA
RANBP9
250
130
100
70
55
50
37
35
25

Fig. S2. Original scans of Figure 3C.

Figure 3D

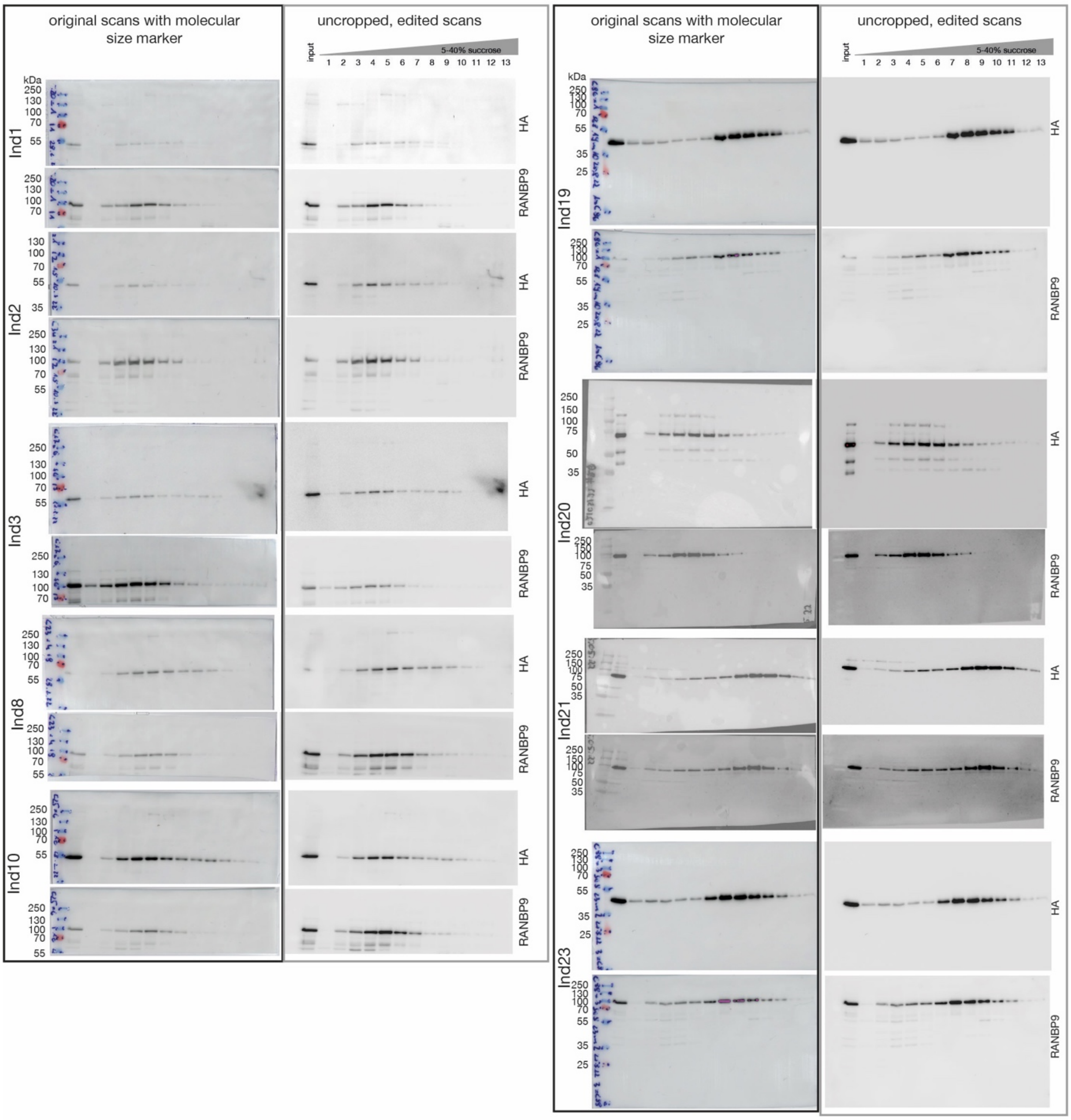

Fig. S3. Original scans of Figure 3D

Figure 4A-D

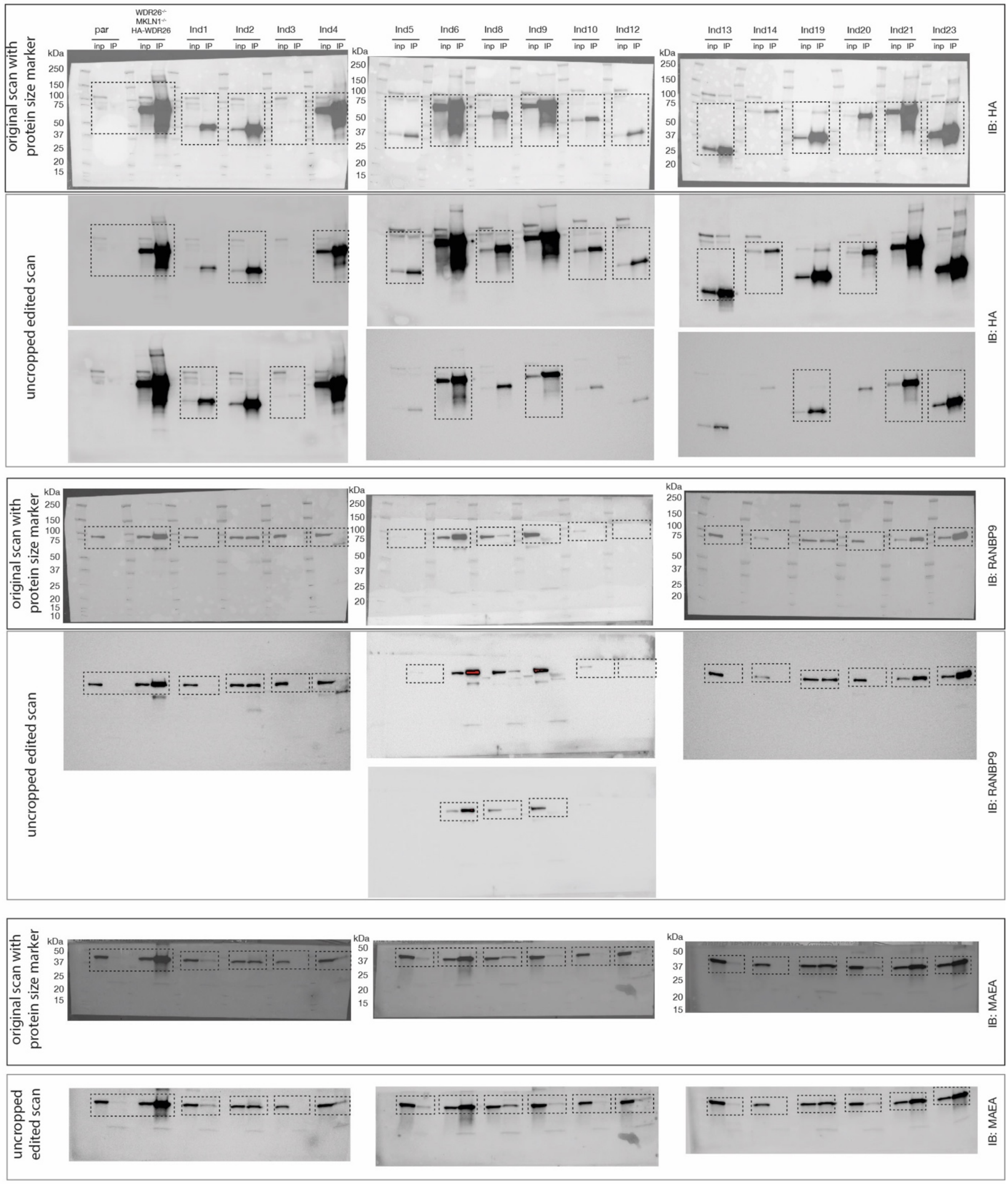

Fig. S4. Original scans of Figure 4A-D.

Figure 5C

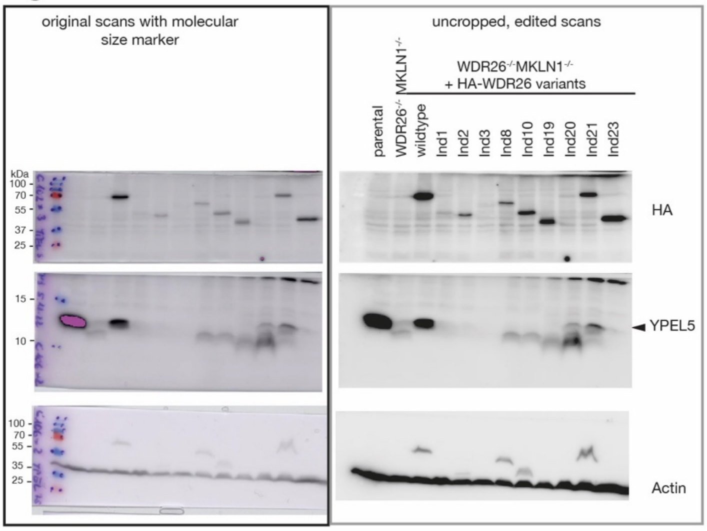

Figure 5D

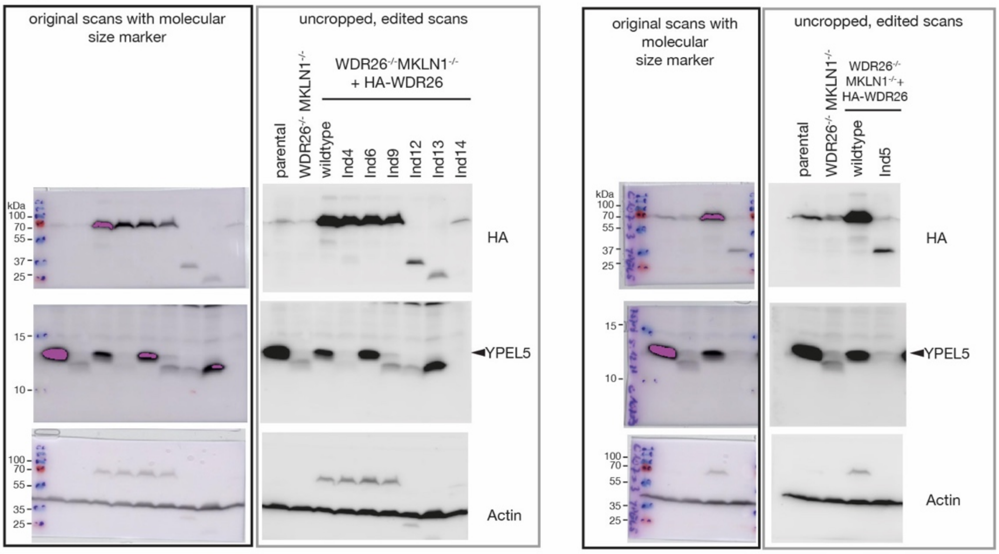

Figure 5F

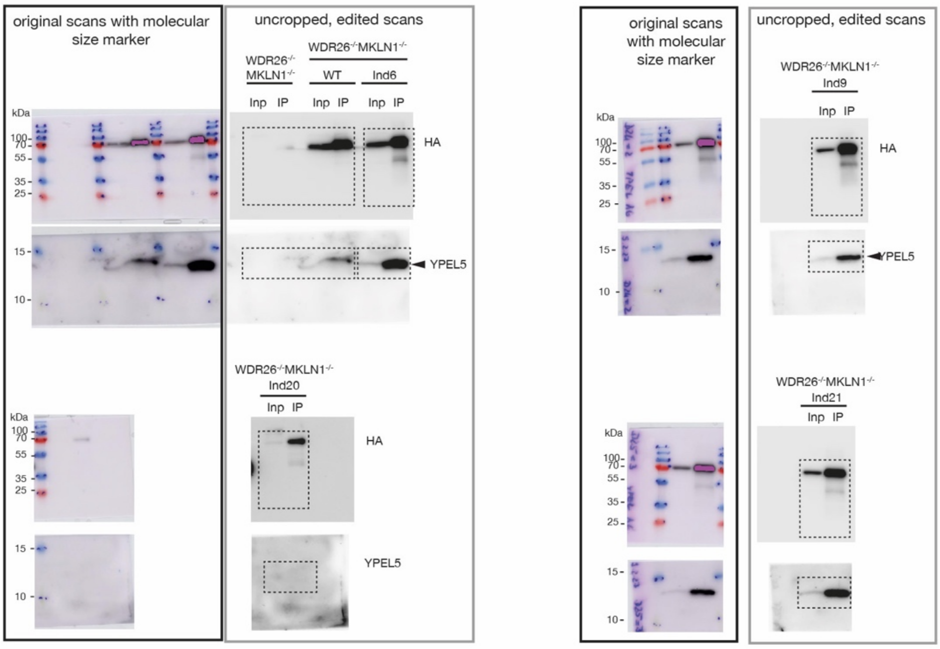

Fig. S5. Original scans of Figure 5C, 5D, and 5F.

Figure 6B

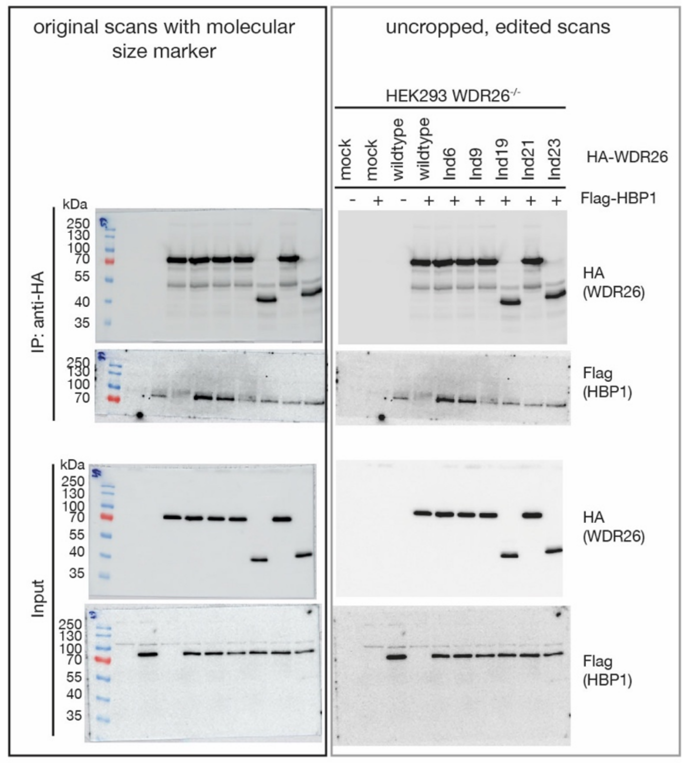

Fig. S6. Original scans of Figure 6B.

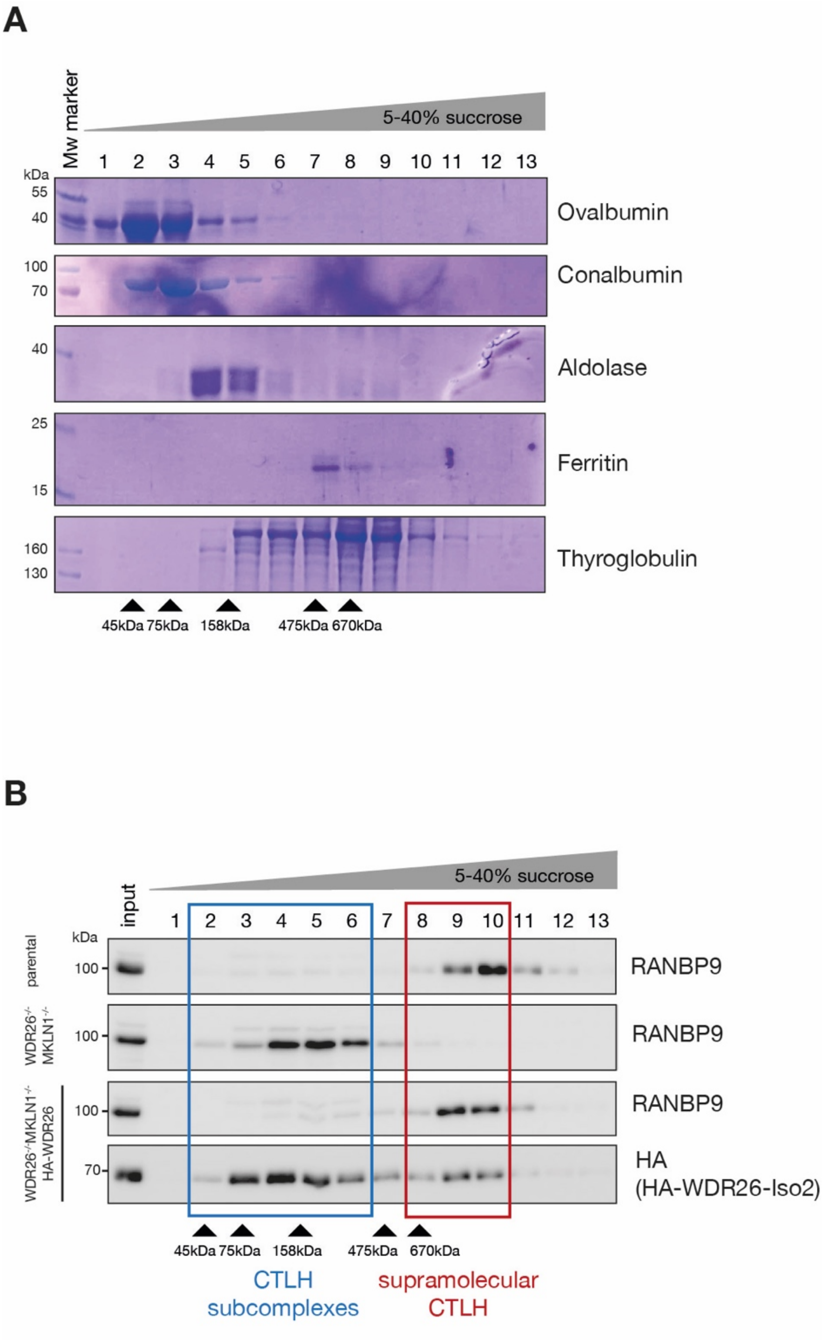

Fig. S7. Analysis of CTLH E3 complex assembly using sucrose density gradient centrifugation. A) Calibration of continuous 5-40% sucrose gradient with indicated proteins, which were fractionated on the sucrose gradient, and fractions analyzed by coomassie-stained SDS-PAGE. B) Cell lysates of K562 parental, *WDR26*- and *MKLN1*-deficient double knockout K562 cells ($WDR26^{-/-}$; $MKLN1^{-/-}$), and $WDR26^{-/-}$; $MKLN1^{-/-}$ cells with stably reintroduced HA-tagged WDR26 isoform 2 were fractionated on a continuous 5-40% sucrose gradient, and fractions analyzed by immunoblotting. Fractions with supramolecular assemblies >670 kDa are indicated with a red box, and smaller subcomplexes with a blue box.

Individual	Genomic position	cDNA notation	Effect on protein	Loci with additional mutation	PMID
1	g.224592155C>A	c.1276G>T	p.Glu426*	no	28686853, individual 1
2	g.224592269_224592270del	c.1161_1162del	p.His389Profs*6	*CDC7, NTNG2*	28686853, individual 2
3	g.224586699delA	c.1457del	p.Val486Glufs*9	no	28686853, individual 3
4	g.224612340A>G	c.644T>C	p.Leu215Pro	no	28686853, individual 4
5	g.224606076_224606077delTG	c.904_905del	p.Gln302Aspfs*22	RNASEH2D	28686853, individual 5
6	g.224607232C>T	c.850G>A	p.Asp284Asn	*ARID1B, ZMYND11*	28686853, individual 6
7	g.224621671G>T	c.137C>A	p.Ser46*	n CHD7, EFHC1, MBD5	28686853, individual 7
8	g.224586291G>A	c.1570C>T	p.Gln524*	INTS12	28686853, individual 8
9	g.224612222A>C	c.762T>G	p.Ser254Arg	no	28686853, individual 9
10	g.224592147C>T	c.1284G>A	p.Trp428*	no	28686853, individual 10
11	g.224588650dupA	c.1419+2dupT	splice site	no	28686853, individual 11
12	g.224607247G>A	c.835C>T	p.Arg279*	TACC3	28686853, individual 12
13	g.224619232dupT	c.574dupA	p.Ile192Asnfs*8	no	28686853, individual 13
14	g.224619392A>T	c.514T>A	p.Trp172Arg	not reported	28686853, individual 14
15	g.224599128_224599138del	c.1149_1158+1del	p.Val384fs; splice site	not reported	28686853, individual 15
16		c.1644 +1G c.1644 +1G>C c.1644 + 1G>A	Ile522Metfs*13	not reported	33506510, patient #1
17	g.224621796del	c.12del	p.Asn4Lysfs*71	not reported	33506510, patient #2
18	g.224621542C>G	c.266C>G	p.Ser89*	NR4A3	33506510, patient #3
19	g.224599245C>T	c.1042C>T	p.Gln348*	not reported	33506510, patient #4
20	g.224581634_224581635del	c.1856_1857del	p.Val619Glufs*16	not reported	33506510, patient #5
21	g.224586627G>A	c.1529G>A	p.Arg510Gln	not reported	33506510, patient #6
22	g.224621739dupC	c.69dupC	p.Gly24Argfs*48	not reported	33675273, patient 1
23	g.224599211G>A	c.1076G>A	p.Trp359*	not reported	33675273, patient 2
24	g.224431570C>A	c.534C>A	p. Asp187Glufs*2	not reported	36269129
25	g.224418302del	c.977delA	p.Asn326Ifs*2	not reported	PMC9140611, patient1
26	g.224411567A>G	c.1020-2A>G	p.Arg340Sfs*29	not reported	PMC9140611, patient2

Table S1: List of *de novo* WDR26 mutations from 26 published cases with clinical history.
